# Defining Synphenotype Groups in Xenopus tropicalis by Use of Antisense Morpholino Oligonucleotides

**DOI:** 10.1371/journal.pgen.0020193

**Published:** 2006-11-17

**Authors:** Amer Ahmed Rana, Clara Collart, Michael J Gilchrist, J. C Smith

**Affiliations:** 1 Wellcome Trust/Cancer Research UK Gurdon Institute, University of Cambridge, Cambridge, United Kingdom; 2 Department of Zoology, University of Cambridge, Cambridge, United Kingdom; University of Pennsylvania School of Medicine, United States of America

## Abstract

To identify novel genes involved in early development, and as proof-of-principle of a large-scale reverse genetics approach in a vertebrate embryo, we have carried out an antisense morpholino oligonucleotide (MO) screen in *Xenopus tropicalis,* in the course of which we have targeted 202 genes expressed during gastrula stages. MOs were designed to complement sequence between −80 and +25 bases of the initiating AUG codons of the target mRNAs, and the specificities of many were tested by (i) designing different non-overlapping MOs directed against the same mRNA, (ii) injecting MOs differing in five bases, and (iii) performing “rescue” experiments. About 65% of the MOs caused X. tropicalis embryos to develop abnormally (59% of those targeted against novel genes), and we have divided the genes into “synphenotype groups,” members of which cause similar loss-of-function phenotypes and that may function in the same developmental pathways. Analysis of the expression patterns of the 202 genes indicates that members of a synphenotype group are not necessarily members of the same synexpression group. This screen provides new insights into early vertebrate development and paves the way for a more comprehensive MO-based analysis of gene function in *X. tropicalis.*

## Synopsis

Genome sequencing projects have provided remarkable insights into the expression and regulation of many genes. For some species, such as the invertebrates Caenorhabditis elegans and *Drosophila melanogaster,* it has been possible to assign functions to these genes on a genome-wide scale. For the vertebrates, similar efforts are being made in mouse and zebrafish, but work in the former species is expensive and slow, and the zebrafish experienced a whole genome duplication event, so that some genes may have retained redundant functions. Here, this study uses antisense morpholino oligonucleotides (MOs) to show that the diploid amphibian Xenopus tropicalis provides a powerful alternative species. The authors have designed MOs to target sequences around the initiating AUG codons of 202 genes expressed during early development and confirmed that these function in a specific manner. About 65% of the MOs caused embryos to develop abnormally, and the authors have divided the genes into “synphenotype groups,” members of which cause similar loss-of-function phenotypes. Expression pattern analysis indicates that members of a synphenotype group are not necessarily members of the same synexpression group. This screen provides new insights into vertebrate development and paves the way for a comprehensive MO-based analysis of gene function in X. tropicalis.

## Introduction

The results of genome sequencing projects and the extensive analyses of expressed sequence tags (ESTs) have provided remarkable insights into the expression and regulation of many genes. For some species, and especially for invertebrates such as Caenorhabditis elegans and *Drosophila melanogaster,* it has also been possible to assign functions to these genes on a genome-wide scale. Such approaches have frequently employed traditional genetic approaches [1], but in addition, RNA interference (RNAi) has been used to inhibit gene function in a systematic and high-throughput manner in C. elegans [2,3] and *Drosophila* [4,5], while antisense morpholino oligonucleotides (MOs) have been used in a screen for gene function in the ascidian species Ciona intestinalis [6].

“Reverse genetic” screens of these sorts have the advantages of speed (because one does not have to locate the mutated gene) and economy, and a similar high-throughput approach to the investigation of gene function in vertebrate embryos will be very important for a proper understanding of development and disease. Unfortunately, such an approach cannot easily be adopted in mammalian embryos, except when studying very early stages [7], because the embryos are inaccessible and the abilities of most reagents to inhibit gene function decline as the embryos grow. Zebrafish provide a useful and powerful alternative, and indeed quite extensive MO screens have been carried out in this species [8]. However, the zebrafish is not a tetrapod, and like other teleost fish it underwent a whole genome duplication event between 200 and 450 million years ago [9,10], so that some genes are likely to have retained at least partially redundant functions [11,12].

In this paper we show that the tetrapod species Xenopus tropicalis is a useful alternative model organism. X. tropicalis shares most of the advantages of Xenopus laevis as a model system for studying cellular, molecular, and developmental biology [13,14], and it shows a higher degree of synteny to amniotes than does the zebrafish (see http://www.metazome.net). In addition to these advantages, X. tropicalis is diploid (X. laevis is allotetraploid), its genome has been sequenced (http://genome.jgi-psf.org/Xentr4/Xentr4.home.html), it develops more quickly than *X. laevis,* and it has a generation time of approximately 5 months compared with that of 14 months in *X. laevis.*

The technique of choice for inhibiting gene function in *Xenopus* species involves the injection of MOs [15]. MOs are frequently designed to inhibit translation of the target mRNA but can also be used to interfere with the correct splicing of a target pre-mRNA [16,17]. It is important to note that MOs, like RNAi, do not necessarily remove the gene product of interest completely, so the strategy is referred to as gene “knockdown” rather than “knockout.” However, MOs do offer the opportunity to study the functions of large numbers of vertebrate genes very quickly compared with the generation of genetic null mutants, and indeed a pilot screen in *X. tropicalis,* in which the functions of 26 genes were investigated, suggests that this approach might be fruitful [18].

In this paper we use MOs to inhibit the functions of 202 genes in *X. tropicalis.* We have addressed the specificity of the different MOs, assigned the observed phenotypes to different classes, and, as Niehrs and colleagues have done for gene expression patterns [19,20], subdivided the phenotypic classes into synphenotype groups [21]. Work in *Drosophila* [1] and the zebrafish [22,23] has shown that the classification of phenotypes in this way is fundamental to the analysis of development, allowing the identification of genes that are involved in similar developmental processes and which interact with each other genetically or biochemically, directly or indirectly. The expression pattern of each gene has been analyzed by in situ hybridization and by noting the representation of its associated transcripts in different cDNA libraries. All the data are accessible through a Web-based database, and our data pave the way for a more comprehensive MO-based analysis of gene function in X. tropicalis.

## Results

### Gene Selection and Experimental Conditions

Genes were selected for analysis from the Wellcome Trust/CR-UK Gurdon Institute Xenopus tropicalis database (http://informatics.gurdon.cam.ac.uk/online/xt-fl-db.html) [24]. BLAST searching confirmed one of our criteria that all should be conserved between *Xenopus* and mammals, and in addition, for purposes of comparison, we selected some genes that had been studied previously by mutation, knockdown, or dominant-negative technologies, and some that had not. The former category allows the comparison of phenotypes caused by injection of MOs in X. tropicalis with those obtained by other approaches in other species, including mouse and zebrafish, as well as *X. laevis.* The latter category was divided into one group comprising genes about which at least something is known (for example, that they are members of gene families already known to play a role in development, or have been studied in vitro, or contain a particular functional domain) and another comprising genes that are completely novel. A large proportion of genes identified through genome and EST sequencing projects are of unknown function, so analysis of this second group should provide an idea of what results might be obtained in the course of a screen designed to target the entire X. tropicalis transcriptome.

Unless otherwise stated, MOs were designed to complement sequence between −80 and +25 bases of the initiating AUG codon of the target mRNA (see Materials and Methods). Two different doses of MO were injected for each gene studied. First, like Kenwrick and colleagues [18], who also used *X. tropicalis,* we injected 10–15 ng of our oligonucleotides. However, we also noted that experiments in X. laevis can employ up to 90 ng MO [25] and that the significantly smaller embryos of X. tropicalis contain between a third to half as much RNA as those of X. laevis (unpublished data). Our results also show that 30 ng of a control MO causes no detectable effect on the development of *X. tropicalis,* beyond, in some egg batches, a slight delay in development. In an attempt to strike a balance between eliminating the gene product of interest and not causing non-specific effects, we therefore additionally used a dose of 30 ng MO in our experiments.

### Overview

Embryos were examined at early to mid-gastrula (stage 10.5–12), tailbud (stage 22–28), and tadpole (stage 37–41) stages (Figure 1A–1C), and any deviations from normal development at each stage were noted (see below). MOs were modified by the addition of Carboxyfluorescein or Lissamine at their 3′ ends, allowing us to ensure that oligonucleotides were distributed evenly throughout the embryo and, to some extent, that similar amounts of MO had been injected into each embryo (Figure 1D–1F). Higher-power examination of the animal pole region of an embryo at the early gastrula stage revealed that fluorescent MOs are present in both the nucleus and the cytoplasm of cells, but are particularly highly concentrated in the nucleus (Figure 1D').

**Figure 1:**
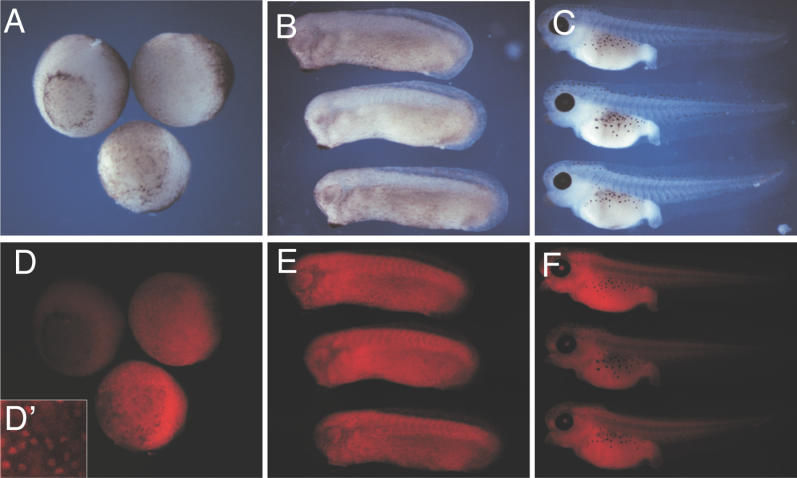
Embryos of X. tropicalis at the Stages Examined for Abnormalities Caused by Injection of MOs Embryos had been injected at the one-cell stage with a Lissamine-labeled control MO. (A–C) Bright field views. (D–F) Fluorescent views. D' shows a high-power view of cells within the animal hemisphere of an embryo at the early gastrula stage. (A and D) Early gastrula stage 10–11. (B and E) Tailbud stage 28. (C and F) Tadpole stage 41.

Table 1 provides an overview of the results obtained in this screen. For the purposes of this analysis we require that a particular MO should cause at least 50% of injected embryos (*n* ≥ 40) to develop in a similar abnormal fashion if the MO is to be classified as yielding a phenotype. In practice, and as described below, our results show that the results obtained at the two doses of MO used in these experiments are similar, and that the “penetrance” of the MOs is usually uniform and high. For example, of the 135 oligonucleotides that yield a phenotype following injection of 30 ng MO, 96% (127) yielded a phenotype following injection of 10 ng. At this lower concentration, 105 of the 127 (83%) showed 100% penetrance (that is, all the embryos developed in a specific abnormal fashion) and 22 (17%) displayed a phenotype in 50%–99% of cases.

**Table 1:**
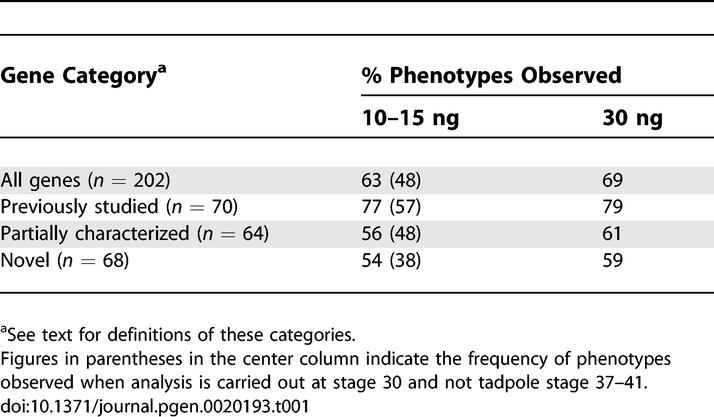
Summary of Results

Together, these observations suggest that a dose of 10–15 ng MO will usually be sufficient to inhibit gene function, although for 4% of the genes screened a phenotype was only observed at the higher dose. In six of these eight cases the penetrance at 30 ng MO was between 80% and 100%.

Each MO was injected on three independent occasions (twice at 10–15 ng and once at 30 ng), and in every case the same phenotype was observed (except in those cases when a phenotype was only observed at 30 ng MO). These observations suggest that the phenotypes are due to gene-specific effects of the MOs and not, for example, to the injection procedure, and that the results are not influenced significantly by genetic variation between the outbred individuals used in these experiments (see Materials and Methods).

The genes studied fell into three classes (see above). For those whose functions have been previously studied, our MOs caused phenotypes in 77% (10–15 ng MO) and 79% (30 ng MO) of cases (*n* = 70; Table 1). For genes that have been partially characterized (that is, they are previously unstudied members of known gene families, or they have only been studied in vitro, or their protein products contain a known functional domain), our MOs caused phenotypes in 56% (10–15 ng) and 61% (30 ng) of cases (*n* = 64; Table 1). Finally, for “novel” genes, we observe a phenotype in 54% (10–15 ng) and 59% (30 ng) of cases (*n* = 68; Table 1).

### MO Specificity: Theoretical and Experimental Considerations

How specific are the phenotypes we observe? At the end of this paper we address this point experimentally for a group of MOs that causes defects in gastrulation, but some general comments are necessary before describing the results we obtain. First, it is unlikely that our MOs exert toxic effects, because injection of 30 ng of a standard control MO has little or no effect on development (see, for example, Figure 2A, 2D, and 2G), and of the 262 MOs injected in the course of this work (some of which are “second site” MOs and excluding the additional MOs, see below), 89 have no effect on development (other than occasionally causing a slight delay) even at the higher dose of 30 ng (Table 1). We also note that MOs that are altered by five bases from their target sequences have little or no effect on development.

**Figure 2:**
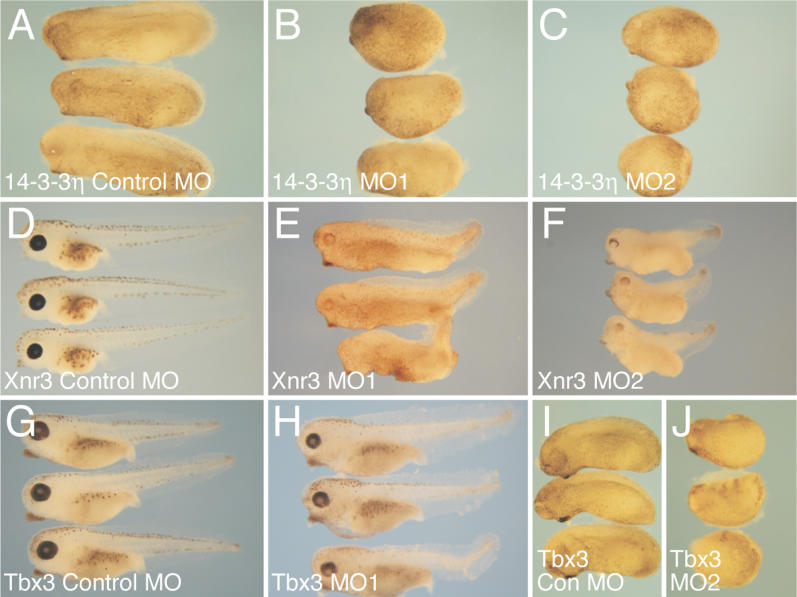
Examples of the Similarities between Phenotypes Caused by Second Site MOs and Those of the Primary MO Directed against Sequence around the Translation Start Site of the Target mRNA (A–C) MOs directed against 14-3-3η. (A) Control MO; embryos develop normally. (B) Embryos injected with MO1, directed against the translation start site of 14-3-3η, develop with a shortened antero-posterior axis. (C) Embryos injected with MO2, directed against sequence 5′ of the translation start site of 14-3-3η, resemble those injected with MO1. (D–F) MOs directed against Xnr3. (D) Control MO; embryos develop normally. (E) Embryos injected with MO1, directed against the translation start site of Xnr3, exhibit an upturned tail. (F) Embryos injected with MO2, directed against sequence 5′ of the translation start site of Xnr3, also have an upturned tail, but they differ slightly from those injected with MO1 because their antero-posterior axes are slightly shortened. (G–J) MOs directed against Tbx3. (G and I) Embryos injected with control MOs develop normally. (H) Embryos injected with MO1, directed against the translation start site of Tbx3, have a normal body axis but their tails are slightly wavy. (J) Embryos injected with MO2, directed against sequence 5′ of the translation start site of Tbx3, have a more severe phenotype than those injected with MO1, in which the antero-posterior axis of the embryo is shortened. MO1, primary MOs; MO2, second site MOs.

In addition, we have asked whether the MOs that are targeted against specific mRNAs complement sequences elsewhere in the X. tropicalis genome. In our experience (see below and also http://www.gene-tools.com), 25-mer MOs that differ in five nucleotides from the target sequence have no effect on the translation of the mRNA in question, so our analysis ignores sequences that differ by more than four nucleotides from a perfect match.

Theoretical calculations based on this criterion and the existing X. tropicalis genome assembly suggest that the probability of there being an additional MO target sequence within the vicinity of the translation start site of an mRNA is as high as 0.3 (unpublished data). This can be extended to allow for the possibility of additional interactions with intron-exon splice sites. If we assume that an “average” gene has seven exons, and that the morpholino must be centered within two or three bases of the splice site to be effective, then this might extend the search space by (say) seven bases per exon. This would add 70 bases of search space per gene to the original 100 bases used around the translation start site, and would therefore increase the probability to ~0.5. We note, however, that the efficacies of MOs with this degree of mismatch are likely to be significantly lower than those of MOs showing a perfect match, and that these efficacies will also depend on factors such as the GC content of the target sequence and the location of the mismatches within the MO. We also note that as the criteria applied become more stringent, the probability of an MO matching an additional sequence elsewhere in the genome becomes much smaller. For example, the probability of there being an additional MO target sequence near the translation start site that differs by up to three nucleotides from a perfect match (rather than four) is approximately 0.003.

Searches for additional target sequences of our MOs by BLAST analyses of the X. tropicalis transcriptome are confounded by the incomplete nature of the genome, but searches of three datasets gave similar results, and the combined results from all three should set a reliable lower limit to the extent of non-specific effects of our MOs. The three datasets were (i) the complete set of Ensembl transcripts for *X. tropicalis,* (ii) the X. tropicalis genome sequence combined with Ensembl/JGI gene models, and (iii) EST clusters that include the complete predicted 5′ UTR. Of the 202 MOs used in this paper, we found that only 14 (6.9%) complement at least 21 out of 25 bases within the 5′ UTR of additional known or predicted open reading frames (Table 2); and of these most differ in three or four rather than one or two bases. For a further 13 MOs we could not decide whether the MO was targeting an additional gene or whether this was the intended target, perhaps obscured by sequencing errors. In the future it will be helpful to have a tool such as AMOD [26] to help in the design of X. tropicalis MOs.

**Table 2:**
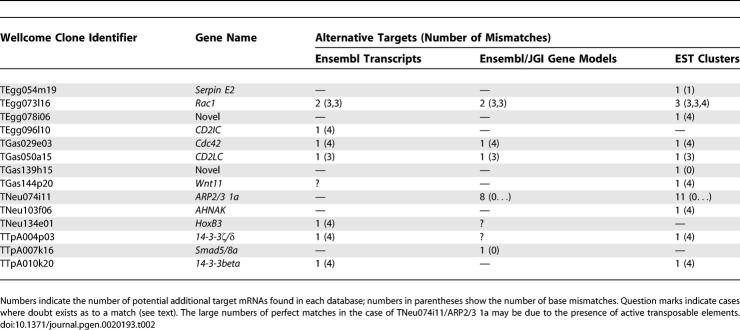
Potential Alternative Targets of the MOs Used in This Paper

It is possible to test the specificity of the phenotype induced by a particular MO experimentally in several ways [17]. One is to perform a “rescue” experiment, in which the MO is co-injected with a form of the target mRNA that lacks the MO-binding site. This approach is labor-intensive, and attempts to rescue the phenotype can often fail even when the phenotype is specific. This may occur, for example, if the targeted gene is expressed in a restricted manner and the presence of the gene product elsewhere in the embryo causes an over-expression phenotype [27]. Successful rescue may also depend on the concentration of rescuing RNA [28]. We have only adopted this approach to investigate the specificities of the MOs that lead to defects in gastrulation (see below).

The second approach is to target the gene in question by means of a second MO that complements a sequence different from that recognized by the first. In this paper, these second site MOs are usually designed to recognize sequence 5′ of the first MO; that is, further upstream in the 5′ UTR. If these second MOs cause a similar phenotype, one can be more confident that this is a specific effect of the loss of the target protein.

Excluding our detailed examination of the specificities of the MOs that lead to gastrulation defects (see below), second site MOs were designed for 48 of the genes which, when targeted by the first MO, resulted in abnormal development. Of these MOs, 34 yielded a phenotype following injection into embryos of X. tropicalis. In most cases (*n* = 21), the phenotypes resembled those caused by the first MO. In others the phenotype appeared to be a less severe version of that caused by the first MO; in only one case (Wnt5b) did the two phenotypes appear strikingly different. Second site MOs were also designed to recognize 12 genes that when targeted by the first MO did not result in a disruption of development. In seven cases no phenotype was observed with the second MO, but five of the second site MOs did cause embryos to develop abnormally (Table 3). As we discuss below, these results, together with the observation that injection of control MOs yields no phenotype, suggest that the effects of our MOs are usually specific.

**Table 3:**
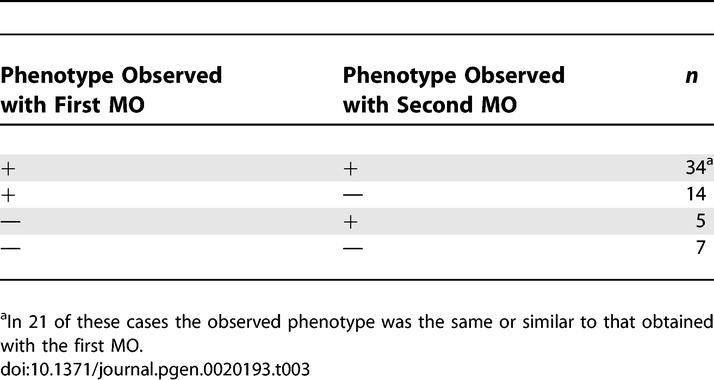
Comparison of Results Obtained with First and Second Site MOs

The final approach is to design MOs whose complement differs from the target sequence in five nucleotides (see http://www.gene-tools.com). This approach has been adopted to investigate the specificities of the MOs that cause defects in gastrulation (see below).

### Expression Patterns, Phenotypic Classes, and Synphenotype Groups

Niehrs and Pollet [20] have introduced the concept of a “synexpression group,” a set of genes that are all expressed in a very similar pattern and are all believed to function in the same developmental process. In this paper, in an analogous manner, we have grouped the various phenotypes we observe, in the hope that this too might help identify genes involved in the same process. A similar approach has been adopted by Chen and colleagues in describing the effects of over-expressing genes in *Xenopus* embryos [21], and as described above, the classification of phenotypes in this way has long been known to be an essential component of forward genetic analyses of development [1,22,23].

Our initial classification divided embryos into seven phenotypic classes based on the time of embryonic lethality, the length and shape of the main body axis, and, for the seventh class, the ability to swim. The members of these classes were then divided into 19 synphenotype groups (Figure 3), each of which comprises genes whose loss-of-function phenotypes resemble each other particularly closely. This exercise may help in coming to understand how the genes function, individually and collectively, in the generation of the early embryo.

**Figure 3:**
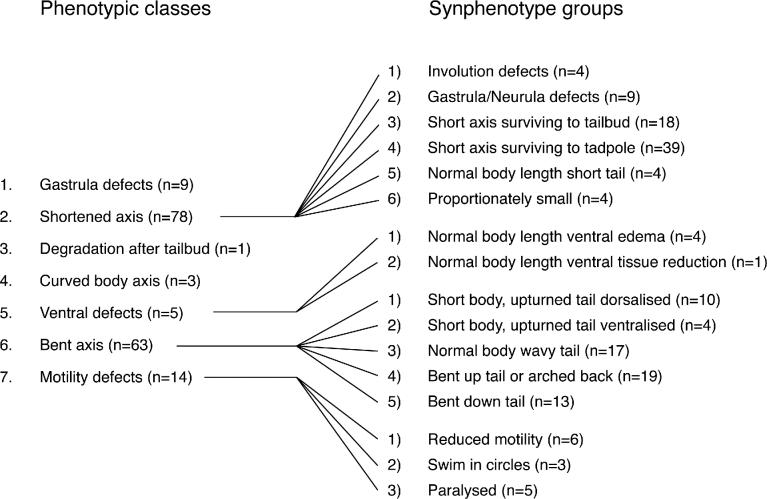
The Phenotypic Classes and Synphenotype Groups Defined by This Work

We describe the seven phenotypic classes and their subdivision into different synphenotype groups, below. These results are summarized in Table 4 and illustrated in Figures 4–13. In some cases a gene that has been targeted by two different MOs may appear in two different groups, because the phenotype caused by the first MO may be more or less extreme than that caused by the second. In other cases it is possible that a gene might be classified as belonging to more than one synphenotype group, and this is indicated in the third column of Table 4.

**Table 4:**
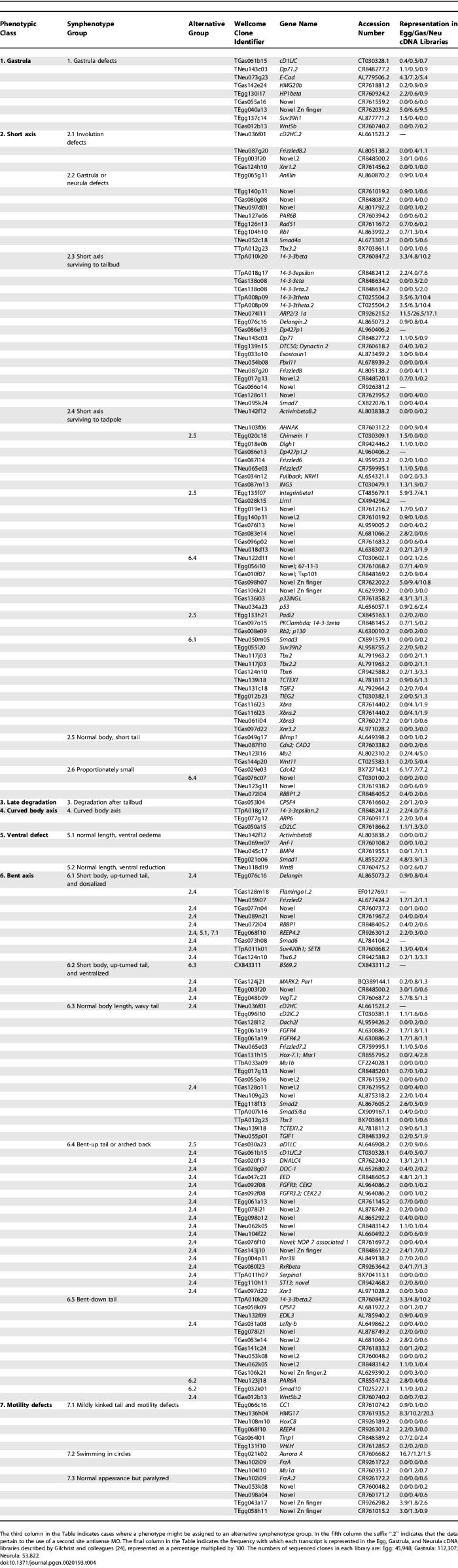
Phenotypic Classes and Synphenotype Groups Identified in This Study

**Figure 4:**
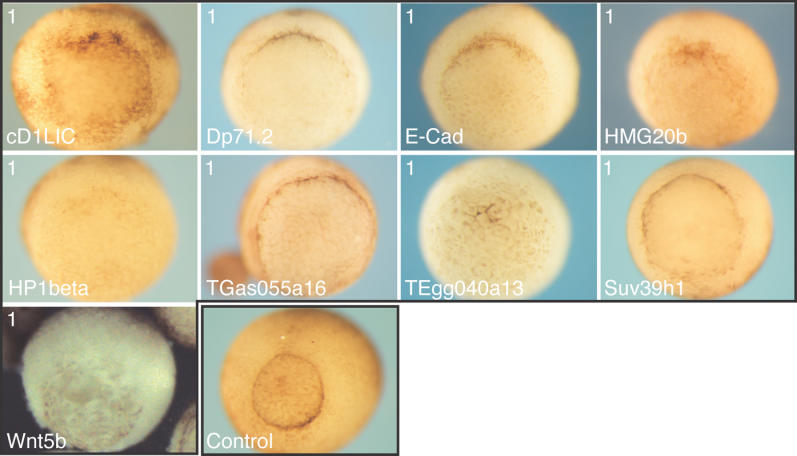
The Nine Members of the Gastrula Defect Phenotypic Class Note that the blastopore in control embryos is closing normally, but is either absent or severely delayed in embryos in which the functions of the indicated genes are inhibited. All embryos shown are at gastrula stage 10.5 to 11.5. In this figure and in Figures 5–13, the number in the top left hand side of each panel represents the synphenotype group to which the embryos belong, and the name of the gene in question is shown bottom left.

**Figure 5:**
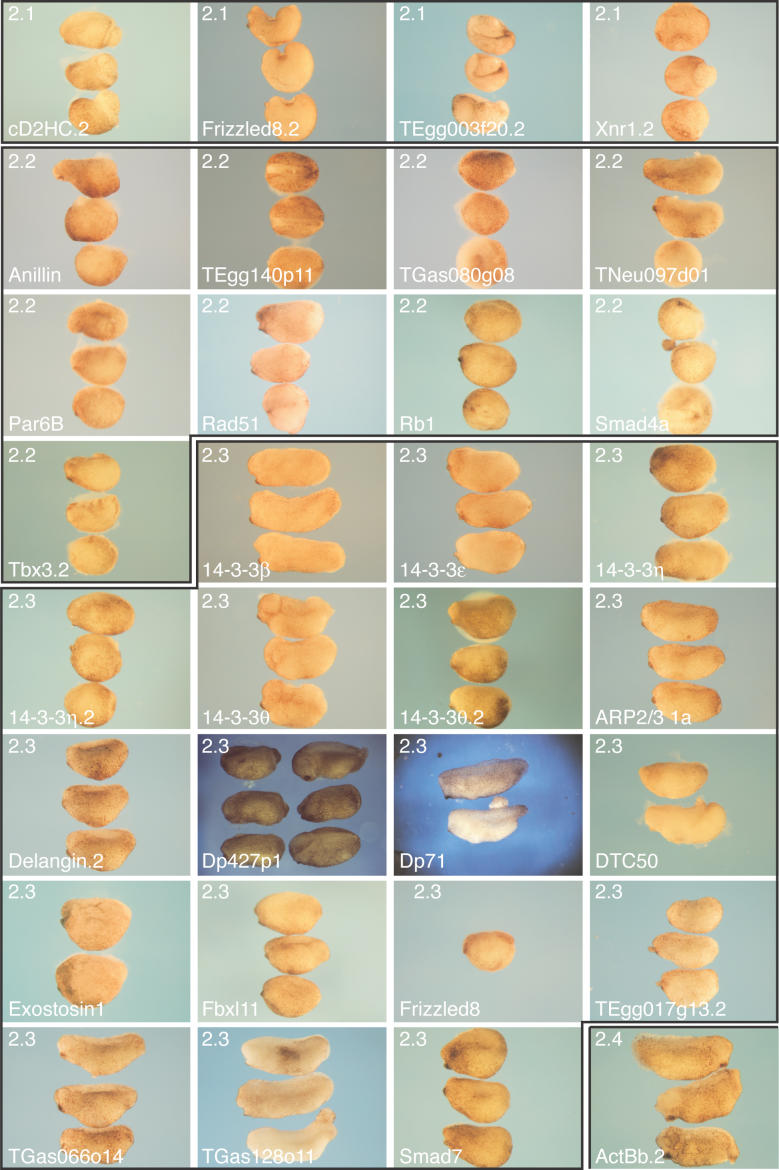
The First 32 Members of the Shortened Axis Phenotypic Class This class can be subdivided into six synphenotype groups, as indicated in Figure 3 and Table 4. Members of the first three synphenotype groups (involution defects, gastrula or neurula defects, and short axis surviving to tailbud) are shown at tailbud stage (stage 24–28), while the member of the second synphenotype group shown here (short axis surviving to tadpole) is shown at tadpole stage 35–41. Lines in this and subsequent figures demarcate the different synexpression groups.

Table 4 also provides an insight into the temporal expression pattern of each gene, by indicating how frequently its transcripts are represented in cDNA libraries derived from egg, gastrula, and neurula cDNA libraries [24]. This is of interest because it is possible that the products of maternally expressed genes are more difficult to deplete than are newly expressed zygotic transcripts [29]. Our analysis, however, reveals no clear relationship between the maternal levels of expression of a gene and the likelihood of an MO directed against that gene producing a phenotype (unpublished data).

**Figure 6:**
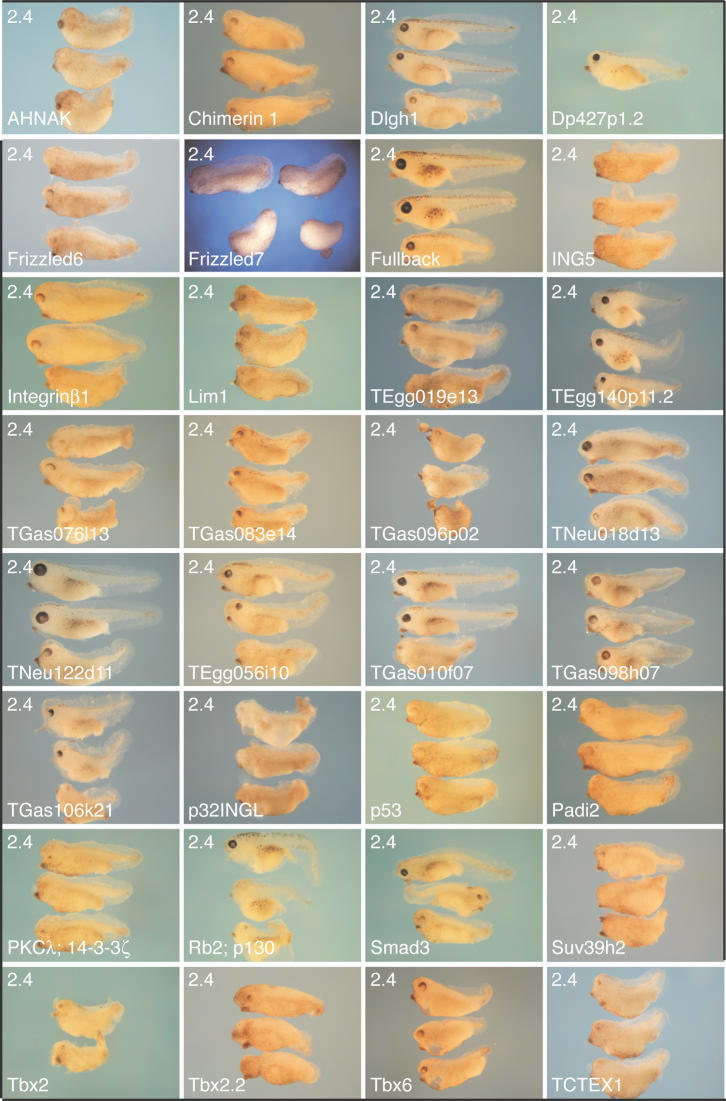
The Second 32 Members of the Shortened Axis Phenotypic Class This class can be subdivided into six synphenotype groups, as indicated in Figure 3 and Table 4. The figure shows examples of the fourth synphenotype group (short axis surviving to tadpole) at tadpole stages 35–41.

### The Phenotypic Classes

#### Gastrula defects.

The members of this class, targeted by nine MOs, exhibit defects during gastrula stages and all die before the mid-neurula stage (Figure 4). The phenotypes falling into this class are very similar, allowing the genes to be classed as a single synphenotype group. The specificities of the MOs that cause these defects are examined in the next section of this paper, and we are currently investigating whether the observed phenotype derives, for example, from defects in germ layer specification or in the cell cycle.

#### Shortened axes.

This class comprises 71 genes (targeted using a total of 78 MOs) whose loss-of-function phenotypes are characterized by a shortening of the anterior-posterior body axis (Figures 5–7). These genes have been divided into six synphenotype groups, including: (i) involution defects (four MOs); (ii) gastrula/neurula defects (nine MOs); (iii) short axis with death occurring after tailbud stages (18 MOs); (iv) short axis surviving to tadpole stages (39 MOs); (v) normal body length with short tail (four MOs); and (vi) proportionately small (four MOs). The observed phenotypes may derive from (among other things) defects in germ layer specification, organizer function, DNA damage, or cell death.

**Figure 7:**
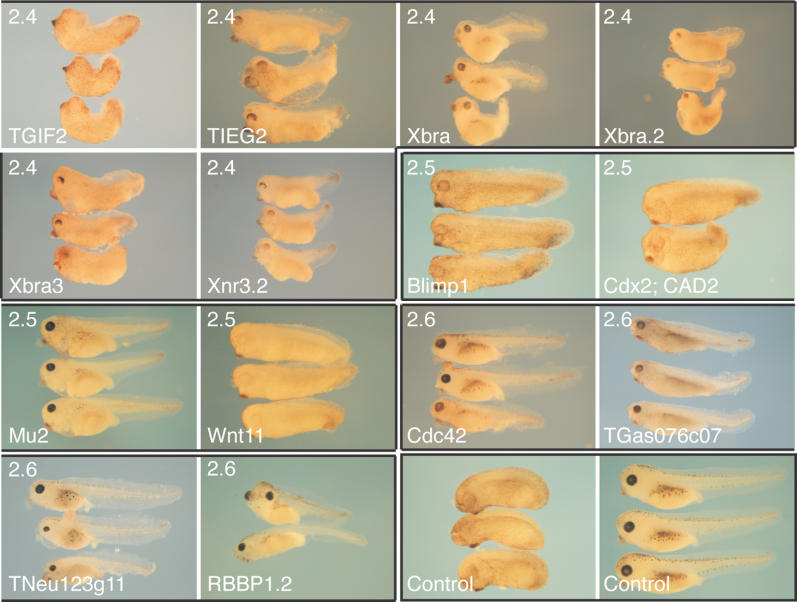
The Final 14 Members of the Shortened Axis Phenotypic Class This class can be subdivided into six synphenotype groups, as indicated in Figure 3 and Table 4. The figure shows examples of the second three synphenotype groups (short axis surviving to tadpole, normal body short tail, and proportionately small) at tadpole stages 35–41.

#### Late degradation.

This class contains only one member: *CPSF4.* Loss-of-function individuals develop normally until the tailbud stage, after which time they begin to disintegrate (Figure 8). This is a more severe phenotype than that observed for zebrafish *CPSF4,* in which null mutants are characterized by a lack of brachial arches [30]. There may be different requirements for the gene in the two species, or perhaps there is some functional redundancy in zebrafish that derives from its partially duplicated genome [9].

**Figure 8:**
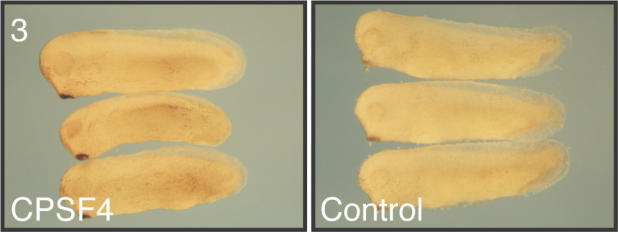
*CPSF4,* the Sole Member of the Late Degradation Phenotypic Class Embryos injected with MOs targeting this gene appear perfectly normal to early tailbud stage 30 but then rapidly disintegrate. Embryos are shown at the tailbud stage (stage 24–28).

#### Curved body axis.

Loss-of-function of the three genes in this class causes the body axes of embryos to curve either to the left or the right (Figure 9). This phenotype may derive from defects in notochord or somites, or in laterality.

**Figure 9:**
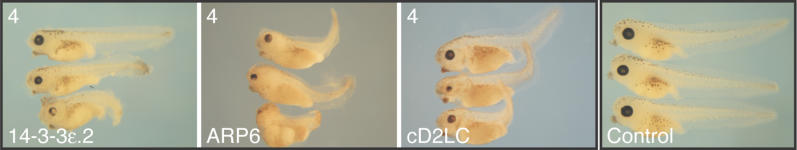
The Three Members of the Curved Body Axis Phenotypic Class Embryos are shown at the tadpole stage (stage 35–41).

#### Ventral tissue defects.

This class contains five genes whose loss-of-function phenotypes are characterized by defects in ventral tissues (Figure 10). Its members can be divided into two synphenotype groups, those with ventral oedema at the tadpole stage (*n* = 4) and those with a reduction in ventral tissue (*n* = 1). The former group may exhibit defects in heart or kidney development, or osmoregulation; the latter may show defects in ventral patterning.

**Figure 10:**
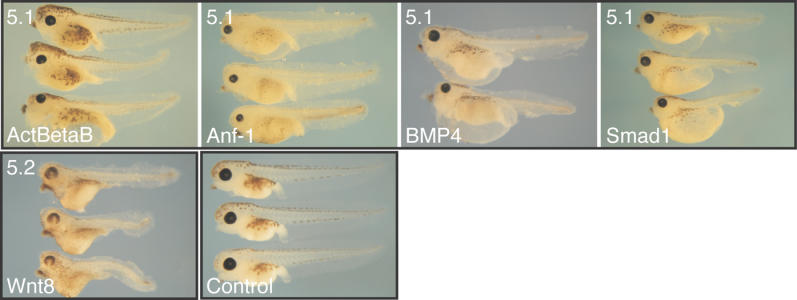
The Five Members of the Ventral Defects Phenotypic Class This class can be subdivided into two synphenotype groups, as indicated in Figure 3 and Table 4. All embryos are shown at the tadpole stage (stage 35–41).

#### Bent axis.

The 61 genes in this class (targeted by 63 MOs) can be divided into five synphenotype groups, all of which are characterized by a bent antero-posterior body axis (Figures 11 and 12). The groups are (i) short dorsalized body with upturned tail (ten MOs); (ii) short ventralized body with upturned tail (four MOs); (iii) normal body and wavy tail (17 MOs); (iv) arched back with bent-up tail (19 MOs); and (v) bent-down tail (13 MOs). All these phenotypes are likely to derive from defects in neural tube, somites, or notochord.

**Figure 11:**
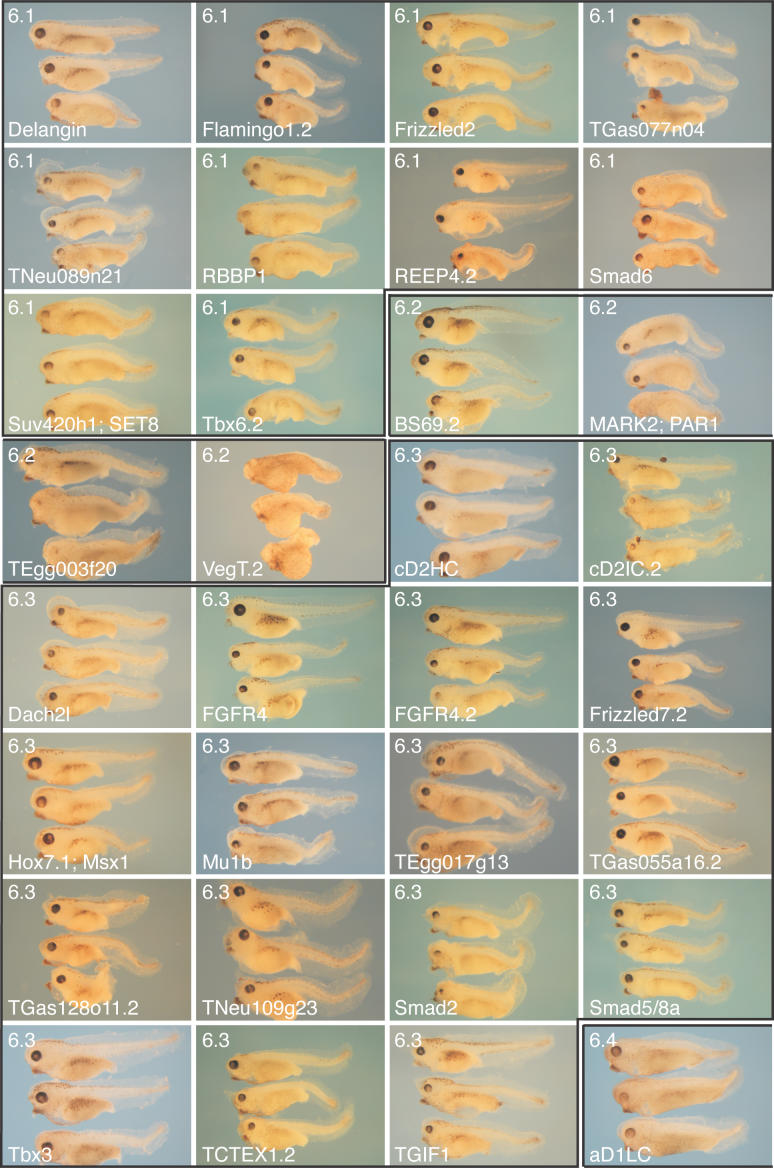
The First 32 Members of the Bent Axis Phenotypic Class This class can be subdivided into five synphenotype groups, as indicated in Figure 3 and Table 4. All embryos are shown at the tadpole stage (stage 35–41).

**Figure 12:**
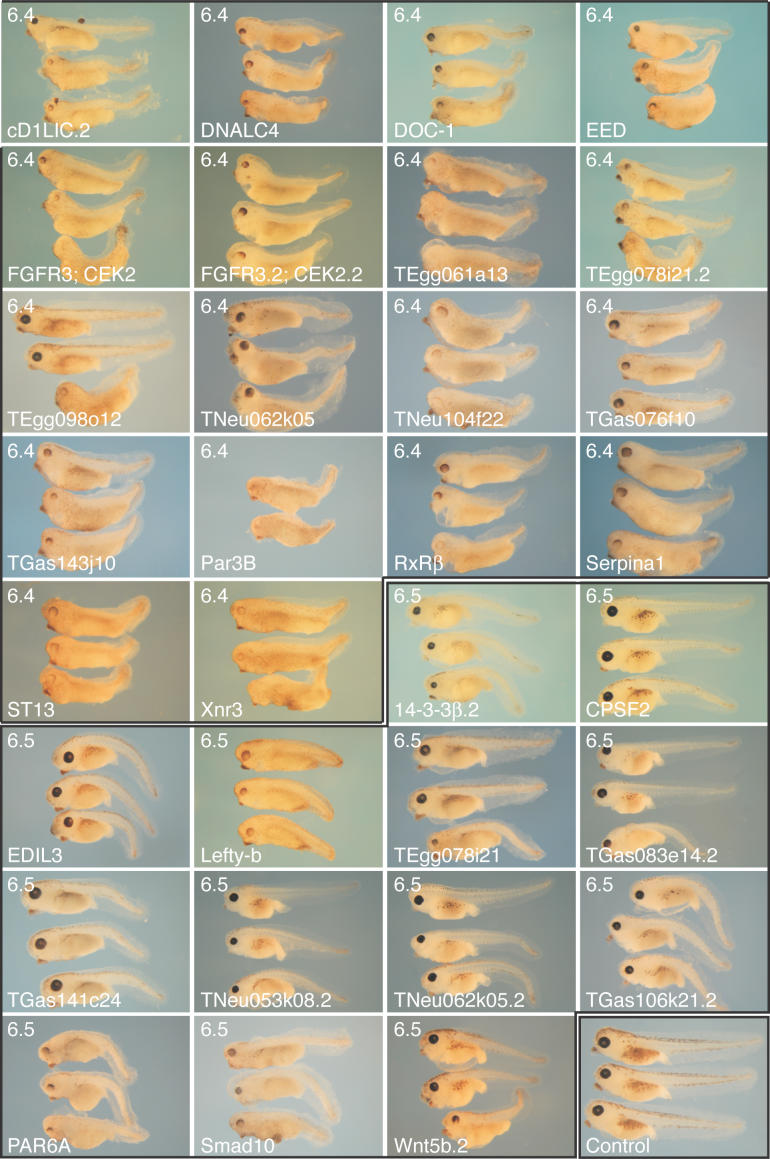
The Final 31 Members of the Bent Axis Phenotypic Class This class can be subdivided into five synphenotype groups, as indicated in Figure 3 and Table 4. All embryos are shown at the tadpole stage (stage 35–41).

#### Motility defects.

This class comprises genes whose loss-of-function causes embryos to develop apparently normally (or perhaps with slight defects in tail development) but whose motility is abnormal, greatly reduced, or absent (Figure 13). The 14 genes in this class can be divided into three synphenotype groups: (i) mildly kinked tail with greatly reduced motility (six MOs); (ii) embryos swim in circles (three MOs); and (iii) embryos are paralyzed and cannot swim even when prodded with a pair of forceps (five MOs). In all these cases, embryonic heartbeat was normal. Effects are likely to derive from defects in muscle or the nervous system, although those that swim in circles may exhibit defects in balance or laterality. This is under investigation.

**Figure 13:**
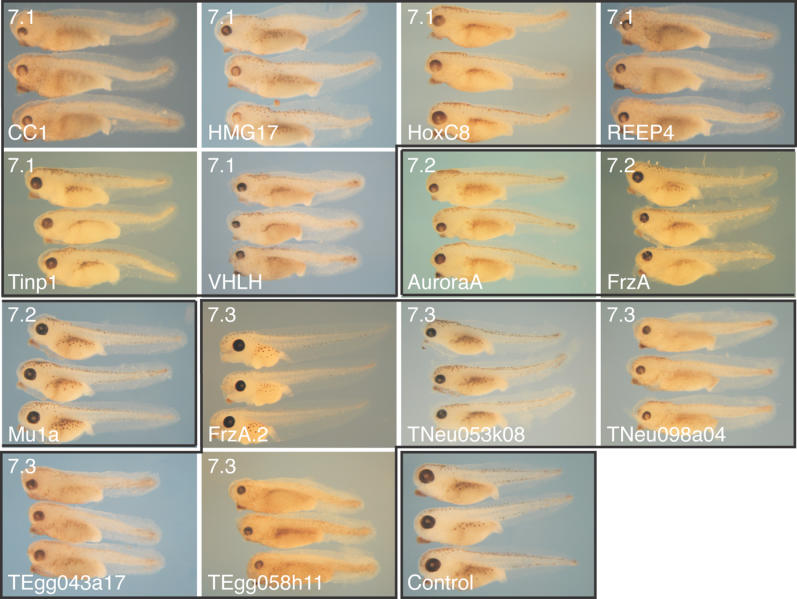
The 14 Members of the Motility Defects Phenotypic Class This class can be subdivided into three synphenotype groups, as indicated in Figure 3 and Table 4. All embryos are shown at the tadpole stage (stage 35–41).

### Specificity Revisited: Apoptosis

As discussed above, interpretation of the experiments described in this paper, and indeed of all experiments making use of MOs, requires that the effects of the MOs are specific. Several criteria for specificity are described above, and these lead us to conclude that the effects of the MOs are usually specific. To investigate this important issue in more detail, we first asked if the possible non-specific effects of MOs might include the induction of apoptotic cell death. Apoptosis might, in particular, underlie later phenotypes such as those exhibited in the “short axis surviving to tadpole” synphenotype group.

To address this possibility we examined embryos from several synphenotype groups using TUNEL staining [31]. Our results (Figure 14) indicate that the Gene Tools standard control MO does not cause an increase in apoptosis at any stage. MOs directed against *BMP4, Flamingo1, Frizzled2, Hox7.1, ING5, p32INGL, Smad1,* and *Tbx6* do not cause significant changes in levels of apoptosis at tailbud stages, even though the MOs in question have usually induced a phenotype by this time. Although MOs directed against *Xbra, Xbra3, Xnr3,* and *Tbx3* do cause a significant increase in apoptosis by tailbud stages, we note that both *Xbra* and *Tbx3* have previously been implicated in programmed cell death [32–34], although the role of Xnr3 in apoptosis remains to be resolved.

**Figure 14:**
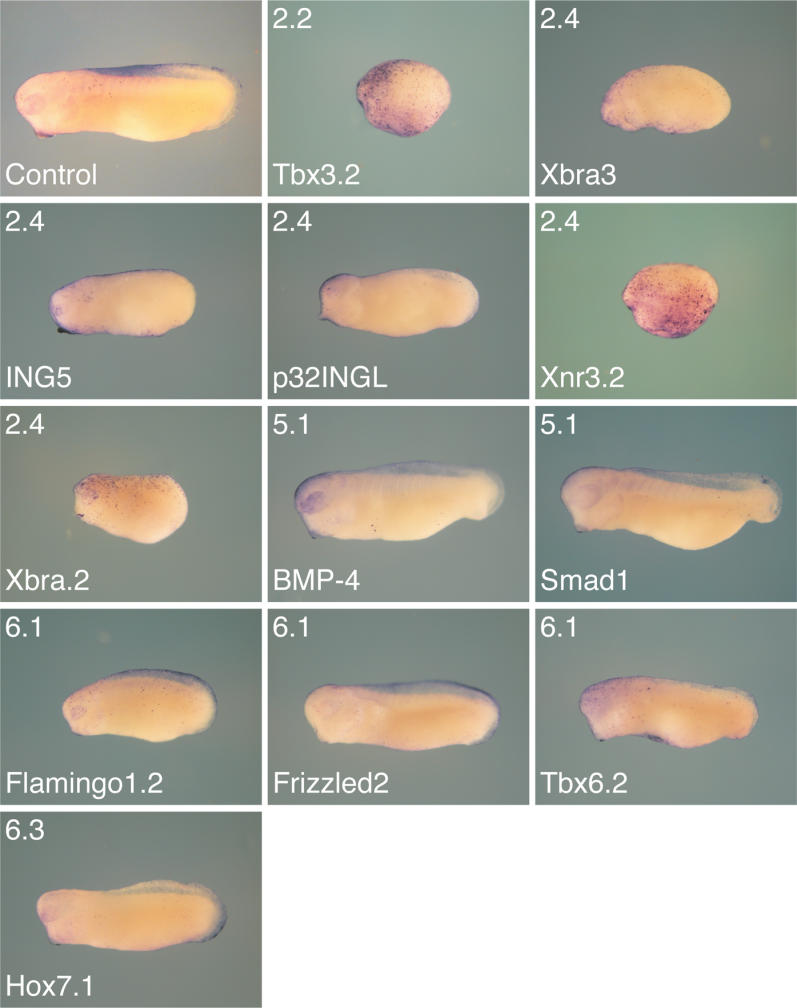
Apoptosis Is Not a Non-Specific Response to Injection of MOs Embryos were injected at the one-cell stage with 15 ng of the indicated MO and allowed to develop to the equivalent of the tailbud stage, when they were examined by TUNEL staining. Note that only the Tbx3.2, Xnr3.2, and Xbra.2 MOs caused a level of apoptosis that exceeded the level observed in control embryos (injected with 30 ng of the Gene Tools control MO).

Together, these results suggest that the phenotypes we observe do not derive from non-specific apoptotic cell death.

### Specificity Revisited: The Gastrula Defects

As a final attempt to investigate the question of the specificity of MOs, we decided to concentrate specifically on the class of gastrula defects, and to ask, for each member of the class, whether a second site MO yields the same phenotype, whether an MO with five altered bases causes a phenotype, and whether the phenotype can be rescued by expression of a form of the target mRNA that lacks sequence complementary to the MO in question. The results of these experiments are shown in Figure 15 and Table 5. They indicate that for all nine MOs that cause gastrula defects, alteration of five bases causes no phenotype to occur in injected embryos, while five of the nine MOs prove to exert specific effects in the sense that the phenotype can be rescued by injection of the cognate mRNA. As discussed above, the latter is a particularly strict test and will underestimate the degree of specificity quite significantly.

**Figure 15:**
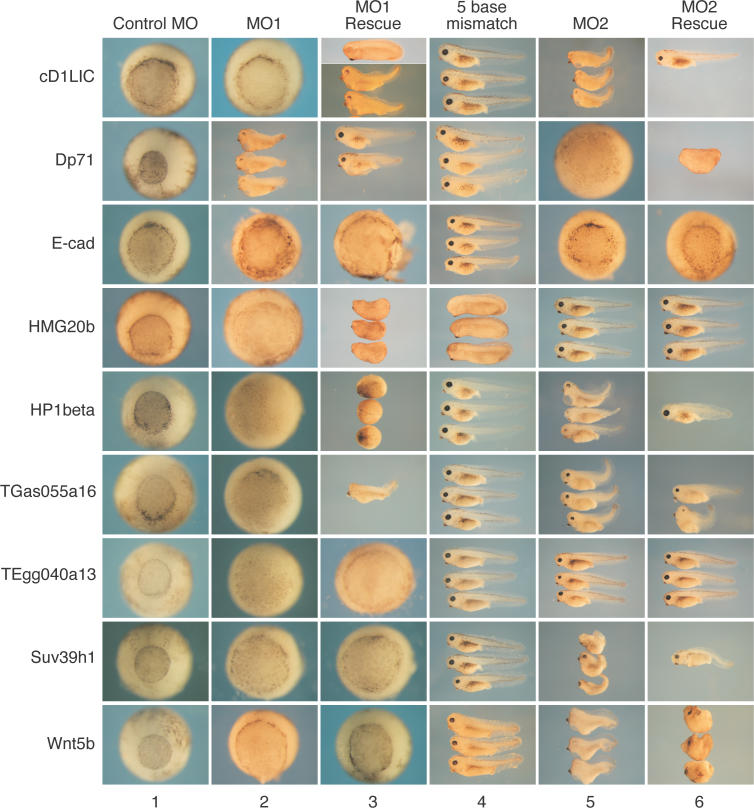
Tests of the Specificities of the Phenotypes Observed in the Gastrula Defects Phenotypic Class The specificities of the MOs used to define this phenotypic class were investigated by injecting 10–15 ng of the Gene Tools standard control MO (Column 1); the original antisense MO (Column 2); MO1 together with 1 ng of a form of the target RNA that lacks the MO target sequence (Column 3); MO1 (or, in the case of Dp71, MO2) with five mismatched bases (Column 4); MO2 (Column 5); MO2 together with 1 ng of a form of the target RNA that lacks the MO target sequence (Column 6). The results of these experiments are summarized in Table 5. In Column 1 (control MO) embryos are shown at the mid-gastrula stage. Embryos in Column 2 (MO1) are at the same stage as those in Column 1, but (with the exception of Dp71) gastrulation is delayed or inhibited. In the case of Dp71, MO1 does not inhibit gastrulation but does cause embryos to develop with a shortened axis. Column 3 indicates that for five of the nine MOs studied, complete or partial rescue of the phenotype was obtained by injection of the cognate RNA. In these experiments, embryos were allowed to develop beyond gastrula stages to tailbud or tadpole stages. In the case of D1LIC, rescue was more complete at tailbud stages (upper panel) than tadpole stages (lower panel). Column 4 shows that for each of the nine MOs, changing five bases caused them to lose the ability to disrupt development. Use of a second site MO usually causes a milder phenotype than is observed with MO1 (Column 5), but the phenotype is usually specific, in the sense that it can frequently be rescued by injection of the cognate RNA (Column 6). MO1, original antisense oligonucleotide; MO2, second site MO.

**Table 5:**
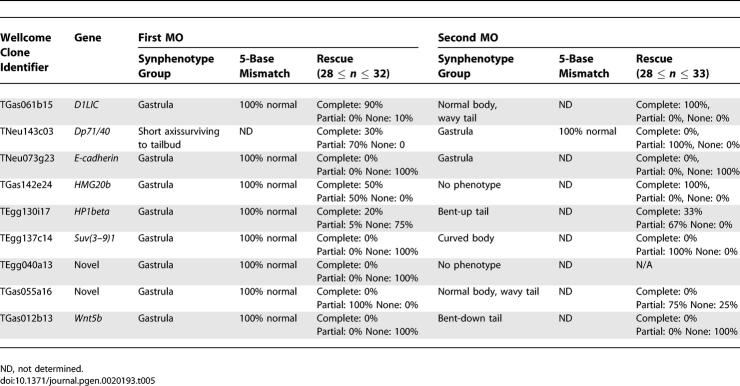
Specificity Controls Applied to the Gastrulation Synphenotype Group

It was possible to rescue the effects of six out of eight of the second site MOs, indicating that their effects too are specific. However, the second site MOs almost invariably yielded a less severe phenotype than did the corresponding first site MOs. This observation is consistent with the results described above (Figure 2 and Table 3), and with the fact that it is the first site MOs that are designed to be the optimum antisense oligonucleotide for the mRNA in question (see Discussion).

Significantly, a less severe phenotype was also observed using second site MOs in a series of experiments investigating the expression of genes such as *Chordin, Xbra, Wnt11, Wnt8, Sox17,* and *E-Cadherin* (unpublished data). In these experiments, injection of an MO with five altered bases caused little alteration in gene expression, while first site MOs directed against, for example, Dp71, HP1beta, and TEgg040a13 caused significant down-regulation of mesodermally expressed genes such as *Chordin, Xbra,* and *Wnt11.* Second site MOs caused much less dramatic down-regulation in the cases of HP1beta and TEgg040a13, consistent with their lack of effect on development (Figure 15), but the second site MO directed against Dp71, which disrupted gastrulation, also interfered with mesodermal gene expression.

### Synphenotype and Synexpression Groups

Genes within a synphenotype group do not necessarily belong to the same synexpression group [20]. This may be illustrated by referring to two synphenotype groups within the class of genes required for normal motility. Thus, of the three genes whose loss-of-activity causes embryos to swim in circles, one *(AuroraA)* is expressed at highest levels in the head (Figure 16A), another *(FrzA)* is expressed most strongly in muscle, heart, pronephros, and otic vesicle (Figure 16B), and the third *(Mu1a)* is expressed in the brain and neural tube but absent from muscle (Figure 16C). Similarly, of the genes whose loss-of-function causes paralysis, one *(TEgg043a17)* is expressed almost ubiquitously (Figure 16D), another (*TNeu053K08)* is highly expressed in muscle (Figure 16E), and yet another *(TNeu098a04)* is most strongly expressed in the posterior neural tube (Figure 16F). These observations are discussed below.

**Figure 16:**
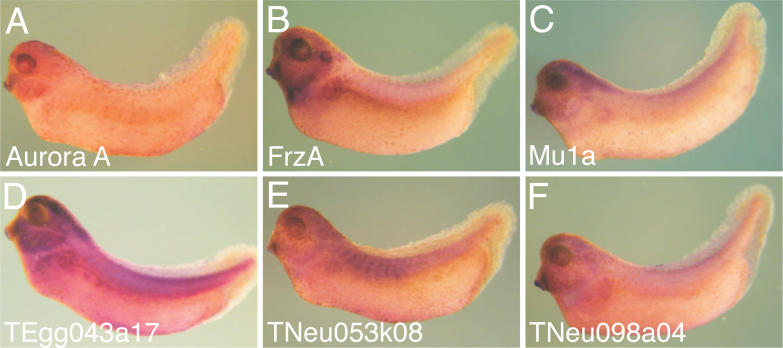
Members of the Same Synphenotype Group Do Not Necessarily Have the Same Expression Patterns The six examples shown here are all from the motility defects class. (A–C) Expression patterns of the three members of the swimming in circles synphenotype group. (D–F) Expression patterns of three members of the normal appearance but paralyzed synphenotype group. Members of each group are not expressed in the same patterns and so do not belong to the same synexpression group (see text).

### Web Access

The results described in this paper can be accessed through a database at http://rd.plos.org/pgen_0057_0001_Xenopus_morpholino_screen. For each gene we have provided (i) the Sanger Institute clone identifier; (ii) the accepted gene name, where available; (iii) MO sequence(s); (iv) phenotypic class and synphenotype group; (v) a description of the phenotype; (vi) images of the phenotype; (vii) images of the expression pattern of the targeted gene; and (viii) the temporal expression profiles of the genes derived from their representation in X. tropicalis cDNA libraries.

## Discussion

In this paper we use MOs to investigate by reverse genetics the functions of 202 genes in *X. tropicalis.* We have assessed the specificities of 60 of the MOs by injecting a second antisense oligonucleotide, compared the functions of the X. tropicalis genes with those of their orthologs in other species (including the mouse), and divided the genes, based on their loss-of-function phenotypes, into seven phenotypic classes and 19 synphenotype groups. As we discuss below, it is possible that members of the same synphenotype group are involved in the same developmental pathway. We have also tested in some detail the specificities of the MOs that cause defects to occur during gastrulation. Together, our results suggest that a large-scale attempt to inhibit the functions of all genes expressed during gastrula stages of X. tropicalis is feasible and that it should shed light on gene function in other organisms.

### Specificity of MOs

Meaningful interpretation of the results presented in this paper requires that our MOs are non-toxic and act in a specific manner. First, as described above, our experiments indicate that injection of antisense MOs does not cause non-specific phenotypes to form, and there is no evidence for non-specific apoptotic programmed cell death in the embryo (Figure 14).

We have addressed the question of specificity by designing additional, non-overlapping MOs against 60 of the mRNAs targeted in this screen. For the 48 genes where the first MO yielded a phenotype (at a dose of 10–15 ng), the second site MO perturbed development in 34 of the cases, and in 21 of these the phenotype was the same or similar (Figure 2 and Table 3). In the remaining 13 cases, our analysis of the gastrula class of defects (Figure 15 and Table 5) suggests that the disruption of development observed using the second site MO might represent a weaker manifestation of that caused by the first MO.

There were 14 cases in which the first MOs yielded a phenotype but the second MOs did not. In these examples, we suspect that the results obtained with the first site MOs are the more reliable. These were designed as the optimal MOs for the mRNAs in question (see Materials and Methods), and as discussed above, it is likely that the second MOs, designed to be non-overlapping with the first while being targeted to a similar region of the mRNA, are less effective, perhaps because of RNA secondary structure (see also Figure 15 and Table 5). In support of this idea, we note that in three of the five cases where the gene had been studied previously, the first MO phenotypes resembled those obtained by other means in *Xenopus* or in other vertebrates. These include *Frz2, Wnt8,* and *Wnt11* (Table 3).

Our experiments investigating the specificities of the nine MOs that cause defects in gastrulation (Table 5 and Figure 15) are also consistent with the idea that MOs are specific in their effects, because oligonucleotides that differ in five nucleotides from their target sequences have no effect, and the effects of the specific MOs can frequently be rescued by injection of the cognate mRNA. We also observe that the effects of our first site MOs are usually stronger than those of the second site MOs, as noted above.

Of the 12 genes for which the first MO yielded no phenotype, the second MO did cause abnormal development in five cases. Of all the phenotypes observed in this study, these, representing just 2% of the total, are perhaps the most likely to be caused by non-specific effects, because the second site MOs may be less effective than the first site MOs in targeting the desired gene product. However, we again note that three of these five genes *(Flamingo1, nodal-related 1,* and *VegT)* have been investigated previously in *Xenopus* or in other vertebrates and that the loss-of-function phenotypes in all three cases resemble those obtained in this study. We suspect, therefore, that the specificity of MOs is, in general, high, and the absence of a phenotype may reflect the complexity of mRNA secondary structure. We conclude that MOs will provide valuable information about gene function in the early embryo of X. tropicalis and will be able to inform future analyses of gene function in other vertebrate embryos.

### Comparison with Other Functional Screens

How does the screen described in this paper compare with others carried out in *Xenopus* and in other species? The most obvious comparison is with a smaller scale X. tropicalis MO screen carried out by Kenwrick and colleagues [18]. These authors targeted 26 genes expressed in the neural plate and neural tube of *X. tropicalis,* and like us required that greater than 50% of injected embryos should develop abnormally for a particular MO to be classified as causing a phenotype. This more limited study reported a lower frequency of loss-of-function phenotypes (23%) than that reported here (62%–67%). However, we note that Kenwrick and colleagues [18] allowed their embryos to develop only to stage 30 and used a dose of MO corresponding to our lower concentration. If we apply the same restrictions to our own larger dataset, using only the first site MOs, we observe loss-of-function phenotypes in 48% of cases (97 out of 202), a figure that is closer to that of Kenwrick et al. [18] and which indicates that many of the phenotypes we observe are detectable only after the tailbud stage. We also note that if we include in this analysis only the 68 novel genes, our frequency of loss-of-function phenotypes falls to just 38% (see Table 1).

MOs have also been used to study gene function in the ascidian Ciona intestinalis [6]. Loss-of-function of 40 of the 200 genes tested in these experiments caused embryos to develop abnormally in ≥50% of cases, and of these genes many had counterparts in mouse and human embryos. At 20%, the frequency of loss-of-function phenotypes in *Ciona* is again lower than that observed in this paper with *X. tropicalis,* although a higher frequency might have been observed if embryos had been allowed to develop for longer or if a higher dose of MO had been used.

Other large-scale screens have been performed in C. elegans and *D. melanogaster.* In *C. elegans,* genome-wide RNAi screens have revealed early embryonic mutant phenotypes in 9% of the genes studied [2,3]; as in our own experiments, it is likely that additional phenotypes would become apparent at later stages of development. In *Drosophila,* RNAi screens have been used to search for novel components of signaling pathways such as the JAK/STAT and Wnt pathways [4,5,35]. These targeted screens inevitably yield fewer phenotypes, but they are very effective in identifying regulators of the signaling pathways in question. A similar targeted screen has also been carried out in the zebrafish, where MOs have been used to study the functions of the homologs of genes expressed in human haematopoietic stem cells. In these experiments, 23% (14/61) of the MOs caused haematopoietic defects in the developing embryos [8].

It is difficult to make comparisons with the frequencies of embryonic phenotypes obtained in mouse embryos, because the mouse data frequently refer to embryonic lethal phenotypes (and many of our milder X. tropicalis phenotypes may not be lethal), and because mutants in which a phenotype is not observed, or which have only a mild postnatal phenotype, are less likely to be published. We note, however, that one study has used a modified gene-trap approach to analyze the functions of 60 genes encoding secreted and membrane proteins and that loss-of-function of one third of these causes embryonic and postnatal death [36]. Another gene-trap study, which more resembles ours in the sense that gene selection was more random, observed phenotypes in 59% of integrations [37], a figure that resembles the 66% observed in the present work.

### Comparisons with Loss-of-Function Phenotypes in Different Species

To ask whether the loss-of-function phenotypes we observe in X. tropicalis might allow us to predict gene function in other species (including mammals), we compared our results with those obtained by the removal of the orthologous gene in other vertebrate species, including the zebrafish and the mouse.

Some of the genes studied here have also been investigated in *X. laevis,* usually by dominant-negative approaches. These include Xbra [38], the FGF receptor [39,40], Frizzled8 [41], Xwnt8 [42], and the BMP receptor [43–45]. In each case the phenotype caused by our MOs in X. tropicalis resembles that observed in *X. laevis,* although the X. tropicalis phenotype is sometimes weaker. For example, the *Xtbra* MO does not produce as severe a shortening of the antero-posterior axis as does the dominant-interfering construct Xbra-En^R^ [38], and the individual MOs targeted against different FGF receptors do not yield phenotypes as dramatic as that caused by the dominant-negative FGF receptor XFD [39,40]. In each case it is likely that the dominant-negative construct is capable of inhibiting the function of more than one gene product. For example, Xbra-En^R^ is likely to inhibit the activities of all three known *Xbra* genes in the X. laevis genome [46–48] while the MOs directed against the X. tropicalis genes will target only one of the two homologs we have identified in that species (Table 4). That said, there are cases where the X. laevis and X. tropicalis phenotypes are very similar. These include, for example, loss-of-function of Frizzled 8, which in both species causes shortening of the antero-posterior axis and defects in neural tube closure [41].

To our knowledge, 57 of the genes analyzed in this paper have been targeted in mouse embryos [49] (Table 6). For 29 of these, the X. tropicalis phenotypes resemble those observed in the equivalent mouse homozygous null mutant and in five cases a phenotype is observed in neither species. For example, loss of *Rad51* function in both X. tropicalis and mouse causes a disruption of gastrulation and neurulation [50] (Figure 5; Tables 4 and 5), while another gene with a conserved biological role proves to be *Cdx2,* which in X. tropicalis is required for proper posterior development and tailbud elongation (Figure 7; Tables 4 and 5). In this case, conventional gene targeting in the mouse embryo does not reveal this function because homozygous null *Cdx2* mutant mice die before implantation [51]. However, tetraploid aggregation experiments show that the gene is also required later in development, during gastrulation and tailbud elongation in a role that resembles its function in frogs [52]. These observations suggest that experiments in X. tropicalis can help reveal the post-implantation functions of genes that cause early lethality.

**Table 6:**
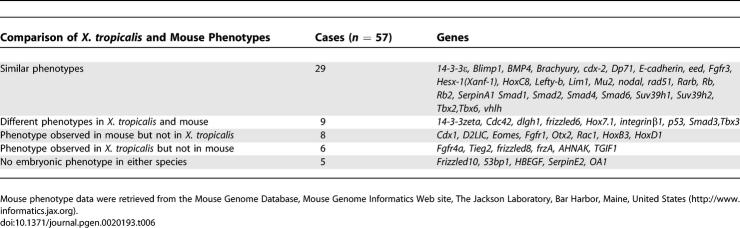
Comparison of Xenopus tropicalis and Mouse Phenotypes

In nine cases, MOs elicited a different phenotype in X. tropicalis and mouse, in eight cases a phenotype has been recorded in the mouse, but was not observed in X. tropicalis in this study, and for six genes a phenotype was observed in frog but not in mouse. These differences may reflect different developmental strategies in the two species, including the deployment of different gene family members to accomplish a specific developmental task, or, particularly for the last group of six genes, functional redundancy in the mouse embryo. The gene *Fgfr4a* illustrates this point. Two MOs directed against this gene product yielded the same phenotype in *X. tropicalis,* in which the body axis appeared normal but the tail of the tadpole was “wavy.” No such phenotype has been observed in the mouse embryo [53], although the gene is required for muscle regeneration in this species [54]. However, we note that mutations in the related gene *Fgfr1* cause abnormal mesodermal patterning in the mouse embryo, with loss of somites and expansion of axial mesoderm at the expense of paraxial tissues [55].

There are also similarities between the phenotypes we observe in X. tropicalis and those obtained in the zebrafish (Table 7). Of the 20 genes for which data are available for both species [56], 12 phenotypes appear to be similar and in five cases a phenotype was observed in neither species. The remaining three differences between X. tropicalis and the zebrafish may derive from differences in biology, a failure to obtain null mutations in zebrafish, a failure of the X. tropicalis MO to inhibit the gene in question, or the whole genome duplication that occurred in zebrafish [9,10].

**Table 7:**
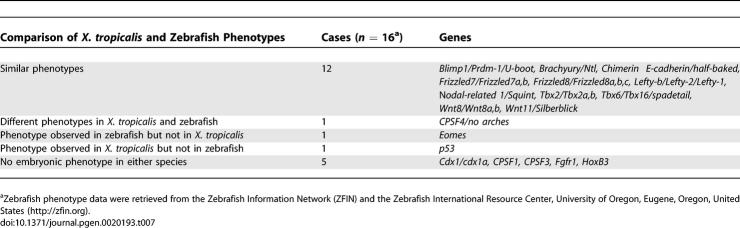
Comparison of Xenopus tropicalis and Zebrafish Phenotypes

Together, the similarities between the phenotypes observed in X. tropicalis and those obtained in other species argues that work in X. tropicalis should shed light on general vertebrate developmental mechanisms.

### Synphenotype Groups and Developmental Pathways

Genetic screens in organisms such as D. melanogaster and the zebrafish Danio rerio allow one to identify genes that function in the same developmental pathway [57], and the same is likely to be true of genes that are members of the same synphenotype groups that are defined in this study. This possibility is illustrated by synphenotype group 6.1 (Short body, upturned tail, dorsalized), which includes the genes *SET8, delangin, flamingo1.2, frizzled2, RBBP1, TBX6.2, Smad6,* and three novel genes. The protein encoded by the first of these genes, SET8, functions as a Histone 4–lysine 20 (H4–K20) methyltransferase. Targeting of this enzyme to heterochromatic regions requires Suv39h1 [58], which itself interacts with pRb [59], which in turn interacts with RBBP, another member of synphenotype group 6.1.

Another more obvious example of genes falling within the same synphenotype group and that function in the same pathway are BMP4, and the molecule that functions downstream of this transforming growth factor type β family member, Smad1 [60]. Significantly, MOs directed against BMP4 and Smad1 can act synergistically, in the sense that simultaneous expression of low doses of BMP4 and Smad1 MOs causes stronger phenotypes than when the oligonucleotides are injected individually (unpublished data).

Although members of a single synphenotype group may indeed be involved in the same developmental or molecular pathway, it is also possible that loss-of-function of different members of a particular molecular pathway may yield distinct phenotypes. Thus, although *RBBP1* is a member of synphenotype group 6.1, its partner *pRb* is required earlier in development and is a member of synphenotype group 2.2 (gastrula or neurula defects). Other members of this group include three novel genes as well as *Anillin, Par6B, Rad51, Smad4,* and *Tbx3,* and it may be significant that the Rb can form a complex that includes members of the Smad family [61].

### The Future

Together, our results suggest that it should be possible to use MOs together with DNA sequence analyses in X. tropicalis [24] to perform a large-scale, low-cost, functional screen in a vertebrate embryo. Such a screen should provide valuable information about gene function in development and disease, and by injecting different doses of MOs it should be possible to study gene dosage effects, thereby creating the equivalent of an allelic series for each gene product. Although in this paper we have not been able to address the efficacy of individual MOs with respect to their ability to depress levels of the targeted protein, this may be possible in the future by use of techniques such as iTRAQ [62] or AQUA [63].

## Materials and Methods

### Embryos and in vitro fertilization.

Adult Xenopus tropicalis were obtained from NASCO (Fort Atkinson, Wisconsin, United States), and are outbred Nigerian frogs derived from University of Virginia stock. Females were primed with 10 U pregnant mare serum gonadotrophin 1–5 d before use and they then received 100 U Chorulon 3–4 h before egg harvesting. Testes were dissected from sacrificed males and macerated in L15 medium containing 10% sheep serum. The sperm suspension was added to eggs and sperm and was activated 3–4 min later by flooding with 0.1× MMR. Embryos were de-jellied using 2% cysteine hydrochloride (pH 7.9–8.1) 8–9 min after flooding and they were then rinsed in 0.01× MMR prior to injection [64].

### MO design and microinjection.

MOs were designed to complement sequence between −80 and +25 nucleotides of the translation start site of the target mRNA. We excluded regions containing polymorphisms by referring to a clustered full-length gene sequence database (http://informatics.gurdon.cam.ac.uk/online/xt-fl-db.html) and then by performing EST searches using the Xenopus tropicalis EST Blast Server at the Wellcome Trust Sanger Institute, Cambridge, United Kingdom (http://www.sanger.ac.uk/cgi-bin/blast/submitblast/x_tropicalis). Morpholinos were designed either by Gene Tools (Philomath, Oregon, United States) or by following the instructions outlined at the Gene Tools Web site (http://www.gene-tools.com). When there were several choices of optimal MO, the sequence closest to the initiating AUG of the target mRNA was selected. Morpholinos were modified by the addition of either Carboxyfluorescein or Lissamine fluorochromes. This allowed us to ensure that all scored embryos had indeed received injections of MOs and that the MOs were uniformly distributed throughout the embryo. For each MO tested, at least 40 X. tropicalis embryos were injected at the one-cell stage with either 10–15 ng or 30 ng of MO at a concentration of 10 ng/nl in H2O. The Gene Tools standard control oligonucleotide 5′-CCTCTTACCTCAGTTACAATTTATA 3′ was used as a control and injected at doses of 10–15 ng and 30 ng.

Samples of the MOs used in this study are available on request while stocks last.

### Specificity and rescue experiments.

To test the specificity of the phenotypes in the gastrulation synphenotype group, embryos were injected with MOs (10–15 ng) that differed in five bases from the target sequence (details available on request). Rescues were carried out by using the PCR to create expression constructs that retain the Kozak sequence of the endogenous mRNA but lack that part of the 5′ UTR which is recognized by MO2. In addition, six base changes were introduced into the region of the mRNA that is recognized by MO1. Details are available on request.

TUNEL analysis was performed as described previously [65]. Embryos were injected at the one-cell stage with 15 ng specific MO or 30 ng of the Gene Tools standard control MO.

### Recording of results.

Embryos were examined and photographed at gastrula (stages 10–12), tailbud (stages 24–28), and tadpole (stages 37–41) stages. Samples were fixed in MEMFA (0.1 M MOPS [pH 7.4], 2 mM EGTA, 1 mM MgSO_4_, and 3.7% formaldehyde) or 4% formaldehyde. For a particular MO to be classified as causing a “phenotype,” we required that its injection should cause at least 50% of embryos (*n* ≥ 40) to develop in a similar abnormal fashion.

## 

## Abbreviations

EST: expressed sequence tag
MO: antisense morpholino oligonucleotide
RNAi: RNA interference
